# Optimized High-Input Practice Enhances Wheat Productivity and Water Use Efficiency by Improving Root Distribution and Canopy Photosynthesis

**DOI:** 10.3390/plants14203176

**Published:** 2025-10-16

**Authors:** Haicheng Xu, Fei Zhao, Yuhai Tang, Qiqin Xue, Jingmin Zhang, Dianliang Peng, Xinglong Dai

**Affiliations:** 1 Shandong Provincial University Laboratory for Protected Horticulture, College of Agronomy, Weifang University of Science and Technology, Weifang 262700, China; 2 College of Health and Human Services, California State University Long Beach, Long Beach, CA 90840, USA; 3 State Key Laboratory of Crop Biology, College of Agronomy, Shandong Agricultural University, Tai’an 271018, China

**Keywords:** agronomic practice, winter wheat, root distribution, canopy capacity, water use efficiency

## Abstract

Integrated agronomic optimization can synergistically enhance crop yields and resource use efficiency. This strategy incorporates suitable sowing date, planting density, and fertilization and irrigation management adapted to the local environment. However, there is a dearth of research on how integrated agronomic optimization practices enhance wheat productivity and water use efficiency (WUE) by improving population root distribution and canopy production capacity. Therefore, a two-year field experiment was conducted in the North China Plain. The experiment involved three integrated agronomic practice treatments with four replications: local farmer’s agronomic practice (FP); high-input agronomic practice (HP), which aimed to explore wheat yield potential regardless of resource input costs; and optimized high-input agronomic practice (OP), which was adapted to local conditions to revamp the wheat production system. Compared to FP and HP, OP involved a later sowing date, higher planting density, and lower N fertilizer or irrigation inputs. Results showed that OP significantly improved grain yield, WUE, N fertilizer productivity (NFP), and net profit compared to FP (*p* < 0.05). Although OP’s yield was 4.25% lower than that of HP, it achieved a 22.99% increase over FP. Compared to HP, OP increased average WUE, NFP, and net profit by 3.08%, 25.68%, and 9.12%, respectively. Over the 2 years, OP promoted deeper roots and higher root length density, which enhanced the uptake of soil water and N. Furthermore, the high transpiration under OP, required for canopy productivity, was sustained by efficient water extraction from deep soil. Additionally, the reduction in unproductive evaporation loss was attributed to increased population density and reduced irrigation. Moreover, OP sustained a higher canopy photosynthetic rate for a longer duration, facilitated by greater post-anthesis N uptake. These improvements in resource acquisition, combined with sustained photosynthetic capacity, ultimately led to more efficient water and N utilization and high grain yield. These indicate that integrated optimization of agronomic practices used under OP can synergistically enhance wheat yield, WUE, and NFP. This was achieved by enlarging and deepening population root distribution while supporting high canopy photosynthesis. Our findings may provide actionable insights into establishing high-yielding, efficient, and profitable wheat production systems in the region.

## 1. Introduction

In the North China Plain (NCP), the winter wheat–summer maize rotation system is the most widely adopted cropping pattern. Their crop production accounted for 57% of domestic wheat and 35% of domestic maize [1]. Over decades, conventional agronomic practices employed by local farmers often led to minimal gains in yield per unit area [2,3], inefficient resource use [4,5], intensified environmental pollution [6,7,8,9], and poor economic returns [10] during wheat production. This was mainly attributed to their early sowing dates, unsuitable planting densities, as well as improper irrigation and excessive fertilization practices [11,12,13,14]. Accordingly, the conventional paradigm of high input and high output is no longer compatible with modern agricultural production. Therefore, exploring the optimized agronomic practices to achieve sustainable wheat production is critically required.

In fact, wheat production is dictated by a multitude of planting management practices. Sowing date, planting density, N fertilization management, and irrigation regulation can directly affect wheat yield and water–fertilizer use efficiency. To address the trade-off between yield and resource efficiency, many studies have explored and validated numerous planting management practices aimed at enhancing wheat yield and resource use efficiency by regulating population quality [15,16,17,18,19,20]. However, few studies have been performed on the response of wheat population productivity and water use to integrated agronomic optimization practices. Here, the integrated agronomic optimization practice is understood as a comprehensive management framework that incorporates suitable sowing date, planting density, and fertilization and irrigation management adapted to the local environment [21,22,23]. We revamped the local wheat production system by formulating an integrated and optimized agronomic strategy. This strategy involves properly delayed sowing, appropriately increased planting density, and modified fertilizer management and irrigation regulation. The better performances in terms of soil quality [14,24], yield formation and N use [2], and radiation use [10] of winter wheat have been achieved. However, there is a dearth of research on whether and how the integrated and optimized agronomic practices enhance wheat productivity and water use efficiency (WUE) by improving population quality.

Clearly, root growth and distribution and canopy production capacity are the two key determinants of population quality. Root distribution is critical driver of soil water absorption and N uptake. Agronomic practices directly affect root growth and distribution. Previous studies have established that appropriately increasing planting density significantly boosts population shoot number, root number, and root length density (RLD) [25,26]. Conversely, delayed sowing resulted in decreased population density and RLD [27]. The capacity of a larger root system to extract more stored soil water increases total water consumption [28]. This enhanced deep-water absorption, in turn, may contribute to maintained canopy production [29]. Moreover, proper irrigation can enhance N utilization [30], and optimal N application can boost water absorption from deep soil [31,32]. Additionally, the availability of water and N governs the processes of assimilate accumulation, translocation, and distribution by influencing canopy photosynthesis and evapotranspiration (ET) [33,34]. Therefore, optimizing root growth and distribution is crucial for boosting wheat yield and WUE. At present, the combined effects of integrated agronomic practices on root–canopy performance remain poorly understood. We hypothesized that the integrated and optimized agronomic practices would enhance wheat productivity and WUE by improving root distribution and canopy photosynthesis.

In the present study, we aimed to (1) explore the effects of integrated agronomic practices on shoot number, root number, and RLD; (2) evaluate the impact of these practices on canopy photosynthesis, stage ET, WUE, the accumulation and translocation of assimilates (including biomass and N), and grain yield; and (3) validate whether and how integrated agronomic optimization practices enhance wheat productivity and WUE. This study is expected to provide valuable insights into further improving local farmers’ agronomic practices to establish a rational wheat population in the target region.

## 2. Results

### 2.1. Shoot Number and Root Number

Over the 2 years, the shoot number per plant was consistently the highest for local farmer’s agronomic practice (FP), followed by high-input agronomic practice (HP), and then optimized high-input agronomic practice (OP, Table 1). Similarly, root number per plant at the jointing and anthesis stages followed the ranking FP > HP ≥ OP. Moreover, the lowest shoot number per unit area was obtained with FP. The number for OP was notably higher than that for HP at anthesis, while the values for both were comparable at jointing. At anthesis, OP exhibited a significantly higher average shoot number per unit area than HP and FP, by 5.37% and 22.02%, respectively (*p* < 0.05). Year, treatment, and their interaction significantly affected root number per unit area (Figure S1A,B). Across all treatments, at anthesis, the root number per unit area averaged across 2 years for OP was 13.02% and 54.21% higher than that for HP and FP, respectively (*p* < 0.05). These results indicate that OP can achieve more shoots and roots per unit area compared to other treatments.

### 2.2. Root Distribution

**Figure 1:**
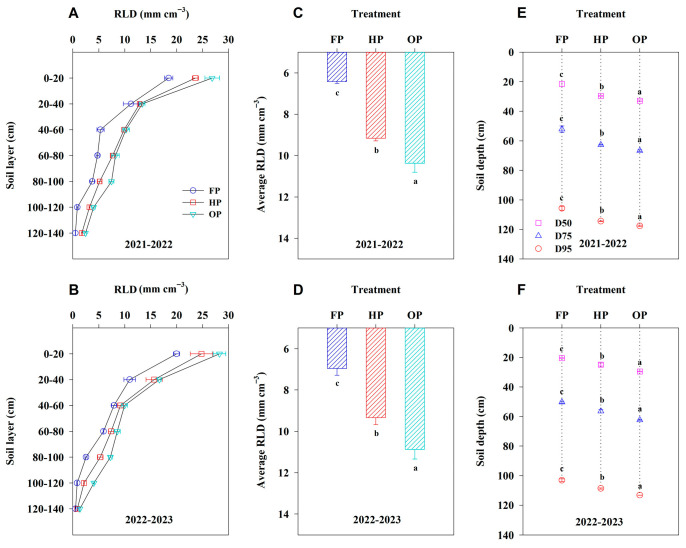
Root length density (RLD) at each 20 cm interval of soil depth, average RLD in the 0–140 cm soil layer, and the depth above which 50%, 75%, and 95% of the roots were located (D50, D75, and D95, respectively) under different treatments in 2021–2022 and 2022–2023. (**A**,**B**), RLD; (**C**,**D**), average RLD; (**E**,**F**), root depth. Error bars represent the standard deviation of the mean. Different lowercase letters indicate significant differences (*p* < 0.05; Fisher LSD test) between treatments.

Over the 2 years, the RLD in each 20 cm soil layer was consistently the highest under OP and the lowest under FP (Figure 1A,B). In the 80–140 cm soil layer, RLD was markedly higher in OP compared to HP, while no notable differences were found between OP and HP in the 20–80 cm layer. OP exhibited a greater average RLD in the 0–140 cm soil layer than HP and FP by 14.81% and 58.84%, respectively (*p* < 0.05, Figure 1C,D). Across all treatments, the depths above which 50%, 75%, and 95% of the roots were located (denoted as D50, D75, and D95, respectively) followed the same ranking of OP > HP > FP (Figure 1E,F). Compared to HP and FP, the OP treatment increased the average vertical root depth by 3.88 cm to 10.99 cm. These findings suggest that OP can achieve a larger RLD and promote a deeper root vertical distribution compared to other treatments.

### 2.3. Soil Water Consumption

**Figure 2:**
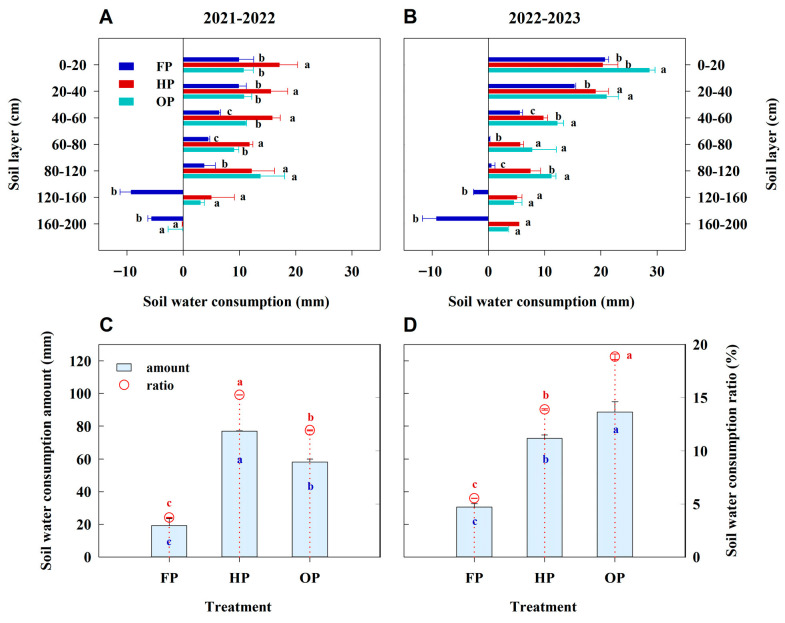
Soil water consumption at each 20 cm interval of soil depth, total soil water consumption in the 0–200 cm soil layer, and soil water consumption ratio during the entire growing season under different treatments in 2021–2022 and 2022–2023. (**A**,**B**), soil water consumption at each soil layer; (**C**,**D**), soil water consumption amount and ratio. Error bars represent the standard deviation of the mean. Different lowercase letters indicate significant differences (*p* < 0.05; Fisher LSD test) between treatments.

Over the 2 years, FP exhibited consistently lower soil water consumption across all soil layers compared to both HP and OP (Figure 2A,B). Moreover, no notable differences in soil water consumption were found between HP and OP in the 120–200 cm soil layer. However, the highest soil water consumption in the 0–80 cm layer occurred under HP in 2021–2022, while it was observed under OP in 2022–2023. Additionally, in 2022–2023, OP had markedly higher soil water consumption than HP in the 80–120 cm layer. Over the 2 years, FP demonstrated the lowest total soil water consumption in terms of amount and ratio (Figure 2C,D). However, we observed inconsistent trends between HP and OP. In 2021–2022, the total soil water consumption amount and ratio followed the same ranking of HP > OP > FP, while the rank order was OP > HP > FP in 2022–2023. These findings indicate that, compared to other treatments, the OP treatment significantly promoted soil water absorption, particularly by facilitating uptake from deeper soil layers.

### 2.4. Stage ET and Stage WUE

**Figure 3:**
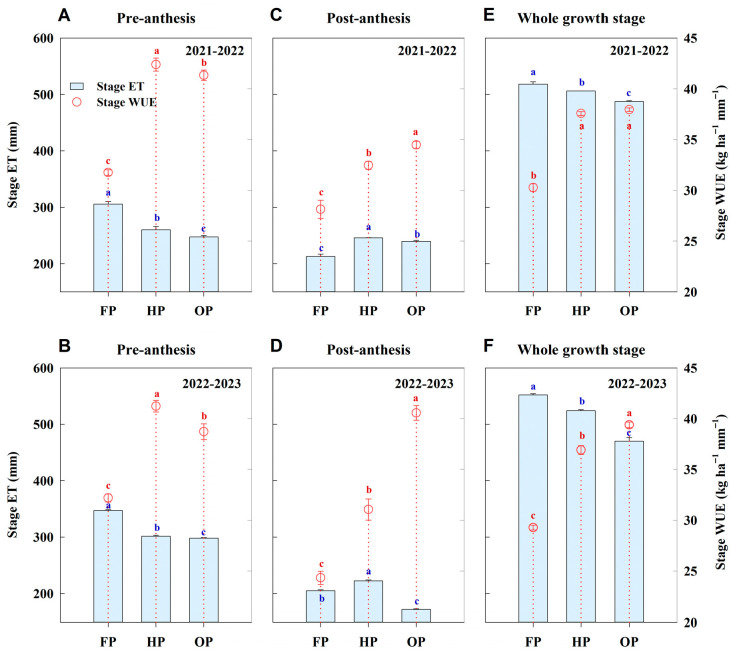
Stage evapotranspiration (ET) and stage water use efficiency (WUE) at the pre-anthesis, post-anthesis, and whole growth stages under different treatments in 2021–2022 and 2022–2023. (**A**,**B**), pre-anthesis; (**C**,**D**), post-anthesis; (**E**,**F**), whole growth stage. Error bars represent the standard deviation of the mean. Different lowercase letters indicate significant differences (*p* < 0.05; Fisher LSD test) between treatments.

FP exhibited the highest pre-anthesis ET and the lowest pre-anthesis WUE (Figure 3A,B). The average pre-anthesis ET was found to be 16.13% and 19.60% higher in FP compared to HP and OP, respectively (*p* < 0.05). However, the pre-anthesis WUE averaged across 2 years for FP was markedly lower than that for HP and OP, by 23.53% and 20.12%, respectively (*p* < 0.05). Additionally, HP exhibited the highest post-anthesis ET (Figure 3C,D). However, the lowest post-anthesis ET was obtained with FP in 2021–2022 but with OP in 2022–2023. Moreover, OP exhibited 18.14% and 43.04% higher average post-anthesis WUE relative to HP and FP, respectively. The total ET over the entire growth period followed the ranking FP > HP > OP (Figure 3E,F). Compared to HP and FP, treatment OP reduced total water consumption by 7.09% and 10.56%, respectively (*p* < 0.05). For the WUE at canopy level, the order was OP ≥ HP > FP.

**Figure 4:**
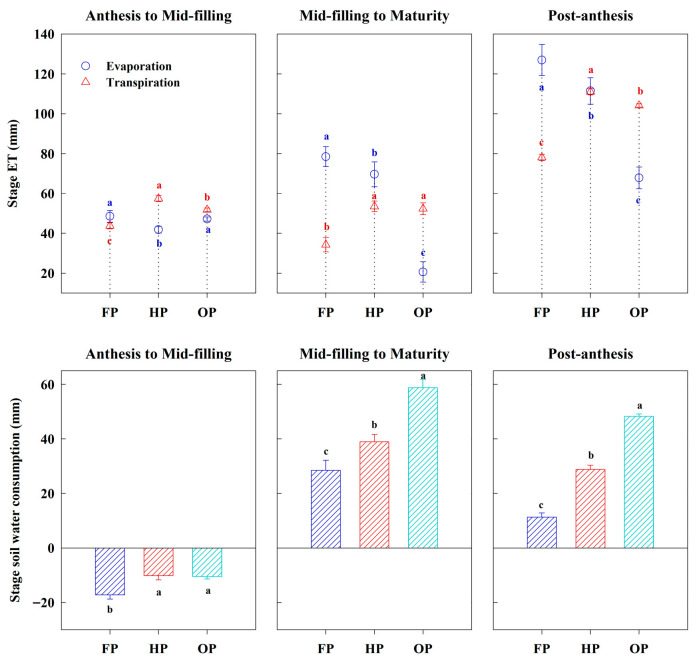
Post-anthesis evaporation, transpiration, and soil water consumption under different treatments in 2022–2023. Error bars represent the standard deviation of the mean. Different lowercase letters indicate significant differences (*p* < 0.05; Fisher LSD test) between treatments.

At the post-anthesis stage, the highest evaporation and the lowest transpiration were obtained with FP (Figure 4). Moreover, the transpiration was the highest for HP, followed by OP. Over the early-to-mid grain filling period, the evaporation for OP was marginally higher than that for HP; however, over the mid-to-late grain filling period, it was notably lower than that for HP. Consequently, over the entire grain filling period, the lowest evaporation was obtained with OP. Furthermore, post-anthesis soil water consumption was highest in OP, intermediate in HP, and lowest in FP. These results indicate that OP treatment achieved efficient use of soil water storage at post-anthesis. This was accomplished by maintaining a high level of transpiration while reducing evaporation loss.

### 2.5. Canopy Apparent Photosynthetic Rate

**Figure 5:**
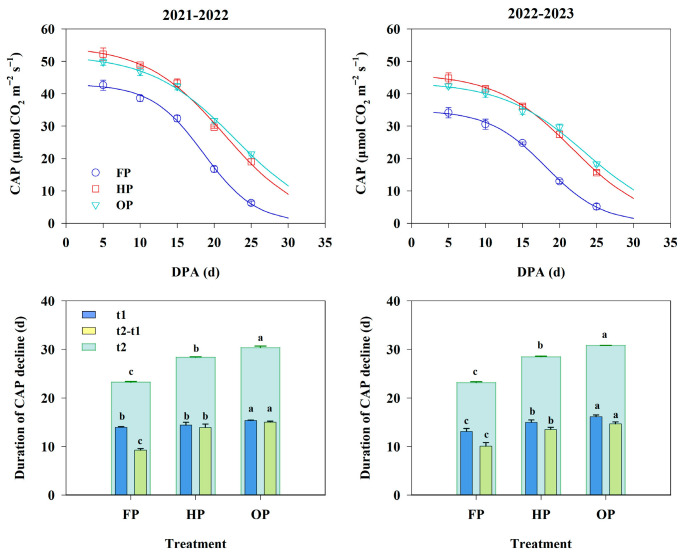
Canopy apparent photosynthetic rate (CAP) and duration of CAP decline under different treatments in 2021–2022 and 2022–2023. t1, the duration of high CAP; t2, the duration of active CAP decline; t2–t1, the duration of rapid CAP decline. Error bars represent the standard deviation of the mean. Different lowercase letters indicate significant differences (*p* < 0.05; Fisher LSD test) between treatments.

The CAP values exhibited a slow–rapid–slow decline tendency with the progress of grain filling (Figure 5). Over the 2-year period, FP consistently exhibited the lowest CAP. During the early-to-mid filling stage, the CAP for HP was marginally higher than that for OP; however, during the mid-to-late filling stage, it was notably lower than that for OP. Based on the logistic model, OP achieved the longest duration of high CAP (t1) and duration of rapid CAP decline (t2–t1). Compared to HP and FP, the duration of active CAP decline (t2) for OP was prolonged by 2.18 d and 7.39 d, respectively. These findings suggest that the agronomic optimization practices employed under OP could promote photosynthetic capacity during the post-anthesis period, especially at the late grain-filling stage.

### 2.6. Grain Yield and Its Source of Assimilates

Over the 2 years, grain yield among all treatments consistently followed the order HP > OP > FP (Table 2). Treatment OP exhibited a 4.25% reduction in average grain yield compared to HP, but a 22.99% increase relative to FP. The pre-anthesis biomass remobilization followed the order HP > FP > OP (Figure S1C). Moreover, the post-anthesis biomass production followed a ranking of OP ≥ HP > FP. Treatment OP exhibited 2.31% and 38.89% higher average post-anthesis biomass relative to HP and FP, respectively. Furthermore, the contribution rate of post-anthesis biomass was highest under OP, intermediate under HP, and lowest under FP. Meanwhile, pre-anthesis biomass remobilization rate exhibited the opposite trend (Figure S1D,E).

### 2.7. Pre-Anthesis N Remobilization and Post-Anthesis N Uptake

The effects of year, treatment, and their interaction on N accumulation in grains were significant (Figure S1F). Over the 2 years, N accumulation in grains followed a ranking of HP > OP > FP (Table 3). The average N accumulation in grains for OP was 8.64% lower than that for HP and 27.30% higher than that for FP. The pre-anthesis N remobilization was the highest for HP, followed by OP, and then the lowest for FP. Moreover, OP exhibited 16.35% and 176.05% greater average post-anthesis N uptake relative to HP and FP, respectively (*p* < 0.05). Over the 2-year period, the contribution rate of post-anthesis N uptake was highest under OP, intermediate under HP, and lowest under FP. Meanwhile, pre-anthesis N remobilization rate exhibited the opposite trend.

### 2.8. Net Profit, WUE, and NFP

**Figure 6:**
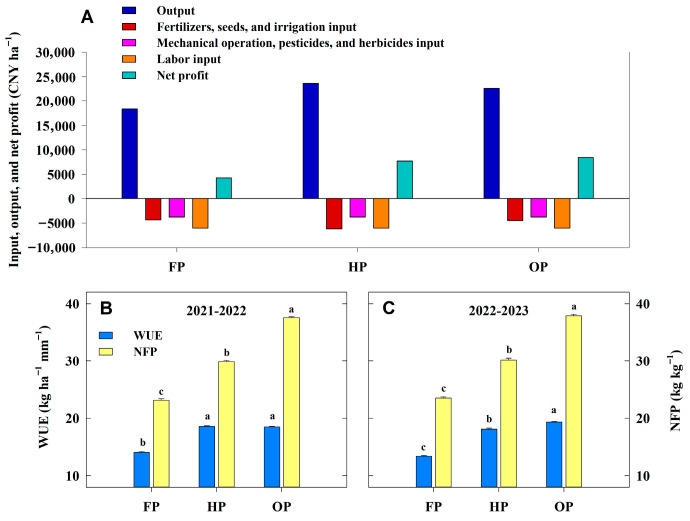
The average economic benefit, water use efficiency (WUE) at yield level, and N fertilizer productivity (NFP) under different treatments in 2021–2022 and 2022–2023. (**A**), input, output, and net profit; (**B**,**C**), WUE at yield level and NFP. Error bars represent the standard deviation of the mean. Different lowercase letters indicate significant differences (*p* < 0.05; Fisher LSD test) between treatments.

The average output across 2 years followed a trend consistent with the average grain yield. The average output for OP was 4.25% lower than that for HP and 22.99% higher than that for FP (Figure 6A). The average costs ranked highest in HP, intermediate in OP, and lowest in FP. The net profit averaged across 2 years was calculated by the average output minus the average costs. Ultimately, OP exhibited 9.12% and 96.61% higher average net profit relative to HP and FP, respectively (*p* < 0.05). Moreover, the average WUE at yield level for OP was higher than that for HP and FP, by 3.08% and 37.47%, respectively (Figure 6B,C). The highest average NFP was also obtained with OP, and was 25.68% and 61.43% higher than that obtained with HP and FP, respectively (*p* < 0.05). These results demonstrate that the OP treatment could achieve higher net profit, higher WUE at yield level, and higher NFP compared to other treatments.

### 2.9. Potential Drivers of Grain Yield and WUE

**Figure 7:**
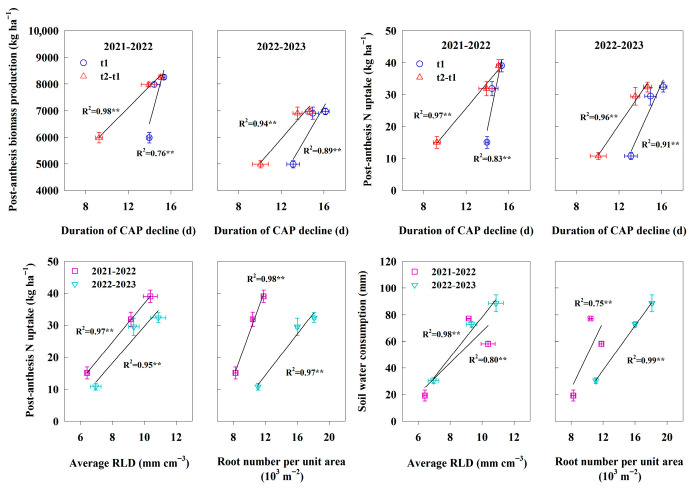
Duration of canopy apparent photosynthetic rate (CAP) decline in relation to post-anthesis biomass production and post-anthesis N uptake, average root length density (RLD) and root number per unit area in relation to post-anthesis N uptake and soil water consumption in 2021–2022 and 2022–2023. t1, the duration of high CAP; t2, the duration of active CAP decline; t2–t1, the duration of rapid CAP decline. **, significant differences at *p* < 0.01 level.

To identify the potential main drivers of grain yield and WUE, we evaluated the main predictors by random forest (RF) analysis (Figure S2). RF analysis showed that, among agronomic practices, sowing date and planting density were both the most important and statistically significant predictors of the changes in yield and WUE (Figure S2A,B). Moreover, among the studied physiological parameters, post-anthesis biomass production, average RLD, duration of active CAP decline, post-anthesis N uptake, shoot number, and duration of high CAP were identified as the significant predictors of yield (Figure S2C). For WUE, the significant variables explaining variations were average RLD, post-anthesis biomass production, duration of active CAP decline, shoot number, and duration of high CAP (Figure S2D). As shown in Figure 7, a significant positive correlation (*p* < 0.01) was found between the duration of CAP decline and both post-anthesis biomass production and N uptake. Average RLD and root number per unit area showed significant positive correlations with post-anthesis N uptake (*p* < 0.01). Moreover, both root number per unit area and average RLD exhibited significant positive correlations with total soil water consumption (*p* < 0.01).

## 3. Discussion

### 3.1. Effects of Integrated Agronomic Optimization Practices on Population Density and Root System

An increase in productive spike number is essential for attaining high yields in winter wheat. Low planting density can increase the shoot number per plant [35], but it may decrease the spike number per unit area, leading to yield loss compared to high planting density [36]. Meanwhile, high planting density means the increased intraspecific competition, which may induce crop yield losses when inadequate control. In this study, FP treatment consistently produced the lowest grain yield mainly due to its lowest shoot (or spike) density. Over the 2-year period, OP treatment increased the average spike number per unit area by 5.37% but reduced the average grain yield by 4.25% compared to the HP treatment. In terms of the modified agronomic practice, OP treatment employed a later sowing date, properly higher planting density, and lower fertilization or irrigation amounts, compared to the HP treatment. Generally, delayed sowing had a similar effect on shoot number as low planting density [27], while it resulted in unchanged or reduced grain yield compared to normal sowing [17,37]. Moreover, grain yield also increased significantly in response to properly increased application rates of N and water [38,39]. Excessive and early inputs of N and water may induce negative impacts. Over the 2 years, the average grain yield for OP treatment was slightly lower than that for HP treatment but significantly higher than that for FP treatment. Our results revealed that a moderate increase in planting density, coupled with optimized N application and irrigation regulation, compensated for the grain yield reduction caused by delayed sowing.

Additionally, root growth and root spatial distribution were closely correlated with population density [40]. Significant increases in root number and RLD were found under appropriately increased planting density and optimized fertilization or irrigation management [25,41]. Conversely, delayed sowing led to a reduction in root growth and RLD [27]. Over the 2 years, integrated agronomic practices significantly affected root number and root distribution. The optimized agronomic practices employed under OP treatment, which included moderately delayed sowing, increased planting density, and refined fertilizer and irrigation management, could achieve higher root number and larger and deeper RLD compared to other treatments. Studies have shown that an enlarged root system often results in the enhanced withdrawal of water stored in the soil [42]. However, over-irrigation may limit the utilization of soil water storage while substantially increasing total water consumption, leading to inefficient water use. Thus, despite the lowest soil water consumption, FP treatment resulted in the highest total water consumption, primarily due to its smaller root system and higher irrigation frequency and amount. Similarly, owing to the lowest irrigation amount and largest root system, the highest soil water consumption was obtained with OP treatment in 2022–2023. However, despite the equal irrigation management and the smaller RLD, the HP treatment resulted in higher soil water consumption compared to the OP treatment in 2021–2022. In other words, the wheat grown under the HP treatment consumed more total water than that grown under the OP treatment.

### 3.2. Effects of Integrated Agronomic Optimization Practices on Stage ET and Stage WUE

Crop water consumption, also known as ET, can be partitioned into transpiration and soil evaporation. We calculated the stage ET and assessed the soil evaporation and transpiration at post-anthesis in 2022–2023. Transpiration is the desired process where water is utilized to sustain plant productivity, while evaporation is considered the undesirable loss of soil moisture from the soil surface [43,44]. The increased crop transpiration and reduced soil evaporation were associated with greater population density and larger canopy size [45,46]. In this study, the lowest population density and two irrigations of the FP treatment promoted the highest soil evaporation and the lowest transpiration, which resulted in substantial unproductive water loss. Moreover, its lowest post-anthesis biomass and high ET directly caused the lowest post-anthesis WUE. The HP treatment exhibited the highest transpiration and relatively high soil evaporation. However, the high water consumption also led to a high ET. Therefore, its highest post-anthesis ET resulted in a lower post-anthesis WUE than that of OP. In the OP treatment, one irrigation at anthesis combined with highest population density effectively minimized non-productive water loss, yielding the lowest soil evaporation. Crucially, compared to the HP treatment, the development of a larger and deeper root system in the OP treatment could enhance the ability to capture deep soil water storage at the late growth stage to maintain a high transpiration level, thereby satisfying canopy productivity, consistent with Zegada-Lizarazu and Iijima [29]. This may be another reason why a steady and high grain yield was observed in the OP treatment. The OP treatment achieved its superior post-anthesis WUE by producing the highest biomass with lowest ET at post-anthesis. In general, ET and WUE are negatively correlated; therefore, lower stage ET will lead to higher stage WUE [47,48]. In our study, OP enhanced post-anthesis WUE not merely by reducing post-anthesis ET, but by shifting it from unproductive evaporation to productive transpiration.

### 3.3. Effects of Integrated Agronomic Optimization Practices on Canopy Production Capacity

The post-anthesis assimilate accumulation fundamentally governs both crop yield and quality [49]. Studies have demonstrated that post-anthesis photosynthetic products can supply 60–90% of the total grain biomass in winter wheat [50]. Over the 2-year period, the contribution of post-anthesis biomass to grain yield ranged from 75% to 84% in our study. The OP treatment produced the greatest post-anthesis biomass and exhibited the highest contribution rate to grain yield. Proper agronomic practices may result in increased photosynthetic rate, prolonged duration of photosynthesis, and delayed leaf senescence [10,51], thereby enhancing biomass production. In our study, OP treatment consistently maintained higher CAP values and achieved longer durations of high CAP and active CAP decline compared to other treatments. The abovementioned results indicate that the wheat grown under the OP treatment could support a high level of canopy photosynthetic capacity during the post-anthesis period. Generally, enhanced crop yield was primarily driven by a more extensive root system, which promoted increased N uptake and greater N accumulation in grains [52,53].

Crop N accumulation at maturity demonstrated a strong correlation with grain yield. Proper irrigation and N application can directly and significantly enhance crop N accumulation [3,30]. However, excessive irrigation leads to both increased inorganic N loss and diminished plant uptake of soil N [54,55]. Moreover, improper N application rates and timing often result in poor N uptake due to a mismatch between N supply and crop demand [56,57]. Furthermore, while appropriately increased planting density is widely known to achieve greater N accumulation [25,26], delayed sowing may weaken N uptake and accumulation [27]. In this study, although FP and HP received the same amount of N fertilizer, FP showed the lowest N accumulation and grain yield, likely resulting from over-irrigation, a lower N topdressing amount, earlier topdressing timing, and smaller population density. Nevertheless, despite lower inputs of irrigation and N fertilizer, along with later sowing, OP achieved significantly higher N accumulation and grain yield than FP. This advantage can be primarily attributed to its higher planting density, which compensated for the detrimental effects of late sowing by increasing the number of shoots per unit area. It is well established that N accumulation in grains relies mainly on pre-anthesis N remobilization [58]. Studies have shown that reduced irrigation and N supply can increase pre-anthesis N remobilization while reducing post-anthesis N uptake [39,59]. This finding was inconsistent with the results observed under our integrated treatments. The integrated optimization agronomic practices employed under OP can significantly enhance post-anthesis N uptake and its contribution rate. Thus, the relative high pre-anthesis N remobilization and highest post-anthesis N uptake of OP resulted in its higher N accumulation in grains compared to FP. This discrepancy may be attributed to the deeper root system. A larger and deeper root system may enhance post-anthesis N uptake, thereby promoting the stay-green effect [60]. The higher post-anthesis N uptake could buffer the pre-anthesis N remobilization from the vegetative organs, thereby preserving the post-anthesis canopy photosynthetic capacity and maintaining the grain yield [61]. In our study, post-anthesis N uptake was highest for OP treatment, intermediate for HP treatment, and lowest for FP treatment. Post-anthesis N uptake was positively associated with the duration of CAP decline, average RLD, and root number per unit area. To summarize, the larger and deeper root system obtained with OP treatment significantly increased post-anthesis N uptake, thereby promoting canopy photosynthetic capacity, which ultimately led to great post-anthesis biomass production and high grain yield.

### 3.4. Integrated Agronomic Optimization Practices Achieved High Yield, Efficiency, and Profitability

Indeed, in this study, the conventional agronomic practices used under the FP treatment resulted in the lower grain yield, inefficient use of water and N, and poor economic benefit. The HP treatment achieved the highest grain yield but had lower WUE at yield level, NFP, and net profit compared to the OP treatment. This was mainly attributed to its excessive resource inputs, particularly the overuse of fertilizers. Our findings indicate that the optimized agronomic practices employed under the OP treatment achieved synergistic improvements in final grain yield, WUE, NFP, and net profit. The better performance of the OP treatment stems from a synergistic interplay between root and canopy processes. Specifically, the larger and deeper root system in OP not only enhanced soil water and N uptake but also supported sustained post-anthesis CAP. Therefore, the improved root distribution directly enabled the enhancement of canopy photosynthetic capacity, which collectively boosted WUE and NFP, and ultimately translated into high yield and profitability. The high profitability of OP was directly attributable to its relatively higher grain yield and lower input costs. This agronomic advantage was underpinned by the following physiological mechanisms: (1) the larger and deeper root system in OP promoted more efficient use of N and soil water, thereby improving WUE and NFP. As a result, OP reduced fertilizer and water requirements without compromising yield, directly lowering the costs. (2) The sustained high CAP at post-anthesis, supported by greater post-anthesis N uptake, was a key driver of the increased biomass accumulation and final grain yield. Therefore, the causal pathway leading to its higher profitability originated from optimized root–canopy performance.

In this study, we focused primarily on determining the comprehensive effects of integrated agronomic practices on wheat performance. Objectively, it is not easy to find out which factor was more effective in improving the performance of wheat. Moreover, the HP treatment obtained higher pre-anthesis ET and higher pre-anthesis WUE at canopy level than the OP treatment. Nevertheless, we only determined the transpiration and evaporation at the post-anthesis stage. In future studies, further in-depth research on the characteristics of canopy transpiration and soil evaporation at each critical stage is needed to elucidate the differences in stage ET. In addition, these results are, however, qualified by the single-site, two-year study. Future studies should be conducted through multilocation trials or long-term monitoring to validate and extend these findings.

## 4. Materials and Methods

### 4.1. Experimental Site

**Figure 8:**
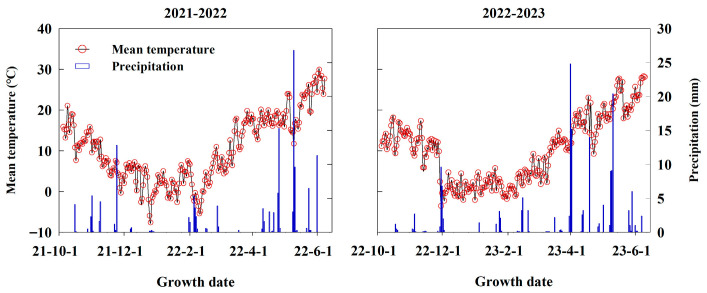
Mean temperature and precipitation recorded in experimental fields during the winter wheat growing seasons from 2021 to 2023.

A 2-year field experiment was conducted during the winter wheat growing seasons from 2021 to 2023, at Beiluo Experimental Station of Weifang University of Science and Technology (36°55′ N, 118°46′ E), located in Weifang, Shandong Province, China. The station experiences a temperate continental monsoon climate, with a mean annual temperature of 13.2 °C, annual sunshine hours of 2491.1 h, and annual precipitation of 612.7 mm. The frost-free period lasts about 200 days per year. Daily mean temperature and precipitation from October to June of the following year are presented in Figure 8. Meteorological data were collected from a local automatic weather station adjacent to the experimental site. Seasonal precipitation during the wheat growing periods reached 149.1 mm in 2021–2022 and 171.5 mm in 2022–2023. The soil is a sandy loam. Prior to sowing in October 2021, the soil characteristics (0–20 cm depth) included soil bulk density 1.42 g cm^−3^, field capacity 28.39%, organic matter 17.08 g kg^−1^, total N 1.15 g kg^−1^, available phosphorus (P) 30.13 mg kg^−1^, and available potassium (K) 88.46 mg kg^−1^. The region follows a winter wheat–summer maize rotation system, with all maize straw from the previous crop incorporated annually into the soil.

### 4.2. Experimental Design

The wheat cultivar ‘Tainong 18’, which is characterized by low tillering capacity and large ears, was cultivated in our experiment due to its widespread adoption in the region. We arranged the following three integrated agronomic practice treatments in a randomized block design with four replications: local farmer’s agronomic practice (FP), high-input agronomic practice (HP), and optimized high-input agronomic practice (OP). The FP treatment followed the conventional agronomic practice of local farmers, including early sowing, low planting density, and improper nutrient management. The FP treatment served as the control. The HP treatment was intended to maximize the grain yields by modifying agronomic practice irrespective of the cost of resource inputs, including proper sowing date, further increased planting density, increased fertilization amount, and reduced irrigation frequency, together with revised timing of fertilization and irrigation, compared to the FP treatment. The OP treatment revamped the local wheat production system by appropriately implementing a later sowing date, higher planting density, and lower fertilization or irrigation amounts, compared to the HP treatment. Each treatment represented a distinct integrated management package.

The combination details for the three integrated agronomic practice treatments are provided in Table 4. Sowing dates were delayed by 5 days from FP (October 5) to OP (October 15). Wheat harvesting occurred on 8 June 2022, and 10 June 2023. Planting densities were 225, 375, and 450 seeds m^−2^ for the FP, HP, and OP treatments, respectively. N, P, and K fertilizers were supplied as Urea (46% *w*/*w* N), calcium superphosphate (12% *w*/*w* P_2_O_5_), and potassium chloride (60% *w*/*w* K_2_O), respectively. Differential fertilizer application rates of N–P_2_O_5_–K_2_O were as follows: 315–120–30 kg ha^−1^ for the FP treatment, 315–210–150 kg ha^−1^ for the HP treatment, and 240–120–75 kg ha^−1^ for the OP treatment. With N fertilizer basal-to-topdressing ratios set at 6:4 (FP) and 4:6 (HP and OP), topdressing was applied at regreening stage for FP and at jointing stage for HP and OP. For all treatments, P and K fertilizers were applied basally. Over the 2 years, FP was irrigated 5 times at the post-sowing, before winter, regreening, anthesis, and mid-filling stages; HP and OP were irrigated 4 times at the post-sowing, jointing, anthesis, and mid-filling stages, with OP having 3-time irrigation at the post-sowing, jointing, and anthesis stages in 2022–2023. Each irrigation amount was 70 mm, as measured by a flow meter. Soil moisture was uniform after irrigation. Each experimental plot (10 × 3 m) contained 12 wheat rows spaced 25 cm apart. In no experimental plot were significant cases of diseases, pests, or weeds observed.

### 4.3. Sampling and Measurements

#### 4.3.1. Shoot Number and Root Number

The total shoot number was recorded within a fixed and marked 1.5 m^2^ quadrat (six rows, 1 m apart) in each plot at both the jointing and anthesis stages. The data were standardized and expressed as the number per square meter. 30 individual wheat plants were sampled from each plot at both the jointing and anthesis stages. We recorded the number of seminal and nodal roots per plant. Following this, we calculated the shoot number per plant and the root number per unit area.

#### 4.3.2. Root Length Density

At anthesis, roots were sampled from a uniform area within a wheat community in each plot. The dimensions of the area were 50 cm in length (spanning two rows of wheat plants and measured perpendicular to the rows), 40 cm in width (measured parallel to the rows), and 140 cm in depth. Each soil core sample was vertically sectioned into 20 cm intervals down to 140 cm in depth. Root samples were placed in string mesh bags and cleaned by rinsing with tap water to remove soil, followed by manual decantation to eliminate organic debris. The roots were picked up quickly by using tweezers, and then dyed immediately with methyl blue solution for at least 12 h. The root samples at each 20 cm interval were scanned separately using a flatbed scanner (HP Scanjet 8200, Hewlett–Packard, Palo Alto, CA, USA). The scanned root images were assessed using a root analysis system (Delta-T Devices Ltd., Cambridge, UK) to determine root length, which was then divided by the volume of the associated soil sample to calculate root length density (RLD, mm cm^−3^). The depths above which 50%, 75%, and 95% of the roots were located (denoted as D50, D75, and D95, respectively) were determined to characterize the vertical RLD distribution [25,62].

#### 4.3.3. Stage Water Consumption

We measured soil moisture at 20 cm intervals from the surface to a depth of 200 cm using a portable time domain reflectometry system (Trime-Pico, IMKO, Ettlingen, Germany), with measurements taken from pre-sowing to post-harvest. Stage soil water consumption was calculated as the difference in soil water storage during a certain growth period. The stage evapotranspiration (ET) was calculated as follows [63]:

(1) Stage ET (mm) = Precipitation amount + Irrigation amount + Soil water consumption during a certain growth period − Surface runoff − Groundwater recharge

Surface runoff was minimized as the plots were bordered by raised beds. Furthermore, groundwater recharge was considered negligible because the water table at the study site was below 8 m.

In accordance with the approach detailed by Yan et al. [64], soil evaporation was assessed at post-anthesis using a small evaporimeter constructed from two PVC tubes. The size of internal tube was 7 cm in inner diameter, 0.5 cm in wall thickness, and 10 cm in height. The external tube, with both an inner diameter and height of 10 cm, was installed within the wheat rows with its upper surface level with the ground. All the soil was removed from the external tube. When taking the undisturbed soil sample, the internal tube was gently pressed into the soil between the rows and the soil at the bottom of the tube was leveled with a cutting knife. The internal tube with soil sample was weighed at 10:00 a.m. of each day using a hundredth electronic balance and then returned to the external tube. The soil samples were replaced after five consecutive days of measurements. Additional measurements were performed after irrigation and precipitation. Each plot included five replicates. In this study, the weight loss of the soil sample per gram corresponded to an evaporation of 0.26 mm. Stage transpiration (mm) was computed by stage ET minus stage soil evaporation.

#### 4.3.4. Grain Yield, Biomass Production, and N Accumulation

At maturity, grain yield (kg ha^−1^) was determined from the total weight of grains harvested from a 3 m^2^ quadrat (comprising six rows spaced 2 m apart) in each plot. Grain samples were air-dried, weighed, and moisture-adjusted to 13% [10]. At both anthesis and maturity, the aboveground part of wheat plants was sampled from two non-border 0.4 m rows of each plot. At anthesis, plant samples were fractionated into stem with sheath, leaf, and spike. At maturity, they were further separated into stem with sheath, leaf, spike axis with glume, and grain. Following oven-drying at 70 °C to constant weight, all fractions were weighed. As described by Zhang et al. [65], dry matter accumulation and remobilization were quantified as follows:

(2) Pre-anthesis biomass remobilization (kg ha^−1^) = Dry matter accumulation at anthesis − Dry matter accumulated in vegetative organs at maturity

(3) Contribution rate of pre-anthesis biomass remobilization to grain yield (%) = Pre-anthesis biomass remobilization/Grain dry weight at maturity × 100

(4) Post-anthesis biomass production (kg ha^−1^) = Grain dry weight at maturity − Pre-anthesis biomass remobilization

(5) Contribution rate of post-anthesis biomass production to grain yield (%) = Post-anthesis biomass production/grain dry weight at maturity × 100

N concentration was measured following the Kjeldahl method [66], and the N accumulation in each plant component was derived by multiplying its dry weight by the corresponding N concentration. N accumulation and remobilization were quantified using the following equations [59]:

(6) Pre-anthesis N remobilization (kg ha^−1^) = N accumulation at anthesis − N accumulation in vegetative organs at maturity

(7) Contribution rate of pre-anthesis N remobilization to N accumulation in grains (%) = Pre-anthesis N remobilization/N accumulation in grains at maturity × 100

(8) Post-anthesis N uptake (kg ha^−1^) = N accumulation at maturity − N accumulation at anthesis

(9) Contribution rate of post-anthesis N uptake to N accumulation in grains (%) = Post-anthesis N uptake/N accumulation in grains at maturity × 100

#### 4.3.5. Canopy Apparent Photosynthetic Rate

The canopy apparent photosynthetic rate (CAP, µmol CO_2_ m^−2^ s^−1^) was measured at 5-day intervals from 5 to 25 days post-anthesis (DPA) with a portable infrared gas analyzer (GXH-3052L, Junfang Institute of Physics and Chemistry, Beijing, China) in each plot. Fabricated from polyester film with 95% light transmittance, the assimilation chamber was 70 cm long, 60 cm wide, and 120 cm high. To ensure proper mixing of air within the chamber, an internal fan with a diameter of 30 cm was installed. Measurements were conducted between 9:30 a.m. and 11:30 a.m. on clear, cloudless days. Prior to each measurement, the chamber was opened to equilibrate the internal CO_2_ concentration with ambient atmospheric levels. 2-row wheat plants were enclosed within the chamber under direct sunlight, and the subsequent rapid decline in CO_2_ concentration was recorded. This decline continued steadily until a drop from approximately 380 to 280 µmol mol^−1^ was sustained for a minimum of 60 s. We calculated the CAP value according to the methods described by Chen et al. [67].

The CAP values for each treatment were fitted, respectively, by a 3-parameter logistic regression analysis as follows [68]:

(10) $$R=\frac{K}{1+{Ae}^{-Bt}}$$

(11) $$R^{\prime }=\frac{dR}{dt}=\frac{KABe^{-Bt}}{{\left(1+{Ae}^{-Bt}\right)}^{2}}$$

where t is DPA; R is the fitted CAP value; K, A, and B are the 3 constants of the logistic model.

We also calculated the second derivative (R″) of Equation (10). The maximum CAP was in accordance with the theoretical value when R″ = 0. Subsequently, we derived the following formulas:

(12) $$t1=\frac{lnA+1.317}{B},t2=\frac{lnA-1.317}{B}$$

where t1 is the starting DPA of a rapid CAP decline that begins from a previously stable high level; t2 is the starting DPA of a slow CAP decline following the rapid CAP decline; t2–t1 is the duration of rapid CAP decline.

#### 4.3.6. Economic Profit, Water Use Efficiency, and N Fertilizer Productivity

The costs include fertilizers, seeds, irrigation, labor, mechanical operation, pesticides, and herbicides inputs. The estimates for output, labor, seed, and fertilizer costs were based on local crop prices, the average daily labor cost, and the average market prices of seeds and fertilizers from 2021 to 2023. The net profit (CNY) averaged across 2 years was calculated by the average output minus the average costs. The water use efficiency (WUE) and N fertilizer productivity (NFP) were calculated by the following formulas:

(13) WUE at canopy level (kg ha^−1^ mm^−1^) = Stage biomass production/Stage ET

(14) WUE at yield level (kg ha^−1^ mm^−1^) = Grain yield/Total ET

(15) NFP (kg kg^−1^) = Grain yield/Fertilizer N amount

### 4.4. Statistical Analysis

All primary data were processed with Microsoft Excel 2013 for preliminary analysis. RLD, soil water consumption, ET, CAP, WUE, and NFP as achieved by the integrated agronomic practice treatments were evaluated by a single-factor analysis of variance (ANOVA). The effects of year and treatment were analyzed by two-factor ANOVA for shoot number and root number, grain yield and N accumulation in grains and their source of assimilates. Statistical methods including ANOVA, logistic regression analysis, and correlation analysis were conducted using DPS 7.05 software (Hangzhou Ruifeng Information Technology Co., Ltd., Hangzhou, China). The least significant difference (LSD) test was used to evaluate mean differences at a significance level of 0.05. We used the “randomForest” package to assess variable importance. The statistical significance of the models and cross-validated R^2^ values were evaluated with the “A3” package via 500 permutations of the response variable. Additionally, we employed the “rfPermute” package to test the significance of each predictor. The generation of figures was performed in SigmaPlot 12.5 (Systat Software, San Jose, CA, USA).

## 5. Conclusions

The lowest grain yield, WUE, NFP, and net profit were consistently obtained with FP. HP exhibited high yield but low efficiency and profitability, mainly due to its excessive inputs. Although the average yield of OP was slightly lower, its average WUE, NFP, and net profit were significantly enhanced compared to those of HP (*p* < 0.05). The integrated agronomic optimization practices (including properly delayed sowing, appropriately increased planting density, and reduced fertilizer and irrigation inputs) employed under OP could synergistically enhance wheat yield, WUE, NFP, and net profit. Wheat grown under OP supported a higher population density and developed a larger, deeper root system. The improvements in the ability of resource acquisition, combined with sustained photosynthetic capacity, ultimately led to more efficient water and N utilization and high grain yield. This study is expected to provide valuable insights into further improving local farmers’ agronomic practices to establish a rational wheat population in the target region. These results are, however, qualified by the single-site, two-year study. Future studies should be conducted through multilocation trials or long-term monitoring to validate and extend these findings.

## Figures and Tables

**Table 1:** Shoot number per plant, shoot number per unit area, root number per plant, and root number per unit area under different treatments in 2021–2022 and 2022–2023.

Year	Treatment	Shoot Number Per Plant		Shoot Number Per Unit Area (10^3^ m^−2^)		Root Number Per Plant		Root Number Per Unit Area (10^3^ m^−2^)	
		At Jointing	At Anthesis	At Jointing	At Anthesis	At Jointing	At Anthesis	At Jointing	At Anthesis
2021–2022	FP	7.75 ± 0.11 a	2.44 ± 0.08 a	1.74 ± 0.02 b	0.55 ± 0.02 c	24.87 ± 0.24 a	36.81 ± 1.03 a	5.59 ± 0.05 c	8.28 ± 0.23 c
	HP	5.41 ± 0.11 b	1.69 ± 0.09 b	2.03 ± 0.04 a	0.63 ± 0.04 b	16.69 ± 0.23 b	27.83 ± 0.33 b	6.26 ± 0.08 b	10.43 ± 0.12 b
	OP	4.37 ± 0.06 c	1.51 ± 0.04 c	1.97 ± 0.03 a	0.68 ± 0.02 a	14.91 ± 0.28 c	26.19 ± 0.59 b	6.71 ± 0.13 a	11.79 ± 0.26 a
2022–2023	FP	8.01 ± 0.51 a	2.61 ± 0.03 a	1.80 ± 0.11 b	0.59 ± 0.01 c	33.80 ± 0.18 a	49.31 ± 0.50 a	7.61 ± 0.04 c	11.09 ± 0.11 c
	HP	5.72 ± 0.13 b	1.82 ± 0.03 b	2.15 ± 0.09 a	0.68 ± 0.01 b	26.88 ± 0.64 b	42.68 ± 0.54 b	10.08 ± 0.24 b	16.00 ± 0.21 b
	OP	4.67 ± 0.01 c	1.57 ± 0.04 c	2.10 ± 0.01 a	0.71 ± 0.02 a	24.55 ± 0.11 c	40.21 ± 0.47 c	11.05 ± 0.05 a	18.10 ± 0.22 a
*p*-value									
Year (Y)		0.0650	0.0001	0.0327	0.0002	0.0001	0.0001	0.0001	0.0001
Treatment (T)		0.0001	0.0001	0.0009	0.0001	0.0001	0.0001	0.0001	0.0001
Y × T		0.9871	0.1689	0.6708	0.5085	0.0879	0.0863	0.0001	0.0001

FP, local farmer’s agronomic practice; HP, high-input agronomic practice; OP, optimized high-input agronomic practice. Values followed by different letters within a column in the same year are significantly different at *p* < 0.05.

**Table 2:** Grain yield and the contribution of pre-anthesis biomass remobilization and post-anthesis biomass production to grain yield under different treatments in 2021–2022 and 2022–2023.

Year	Treatment	Grain Yield (kg ha^−1^)	Pre-Anthesis Biomass Remobilization		Post-Anthesis Biomass Production	
			Amount (kg ha^−1^)	Contribution Rate (%)	Amount (kg ha^−1^)	Contribution Rate (%)
2021–2022	FP	7295.24 ± 88.99 c	1311.03 ± 102.39 b	17.99 ± 1.63 a	5984.21 ± 191.39 c	82.01 ± 1.63 c
	HP	9409.29 ± 47.83 a	1424.21 ± 48.84 a	15.14 ± 0.60 b	7985.08 ± 96.66 b	84.86 ± 0.60 b
	OP	9010.58 ± 43.36 b	748.51 ± 45.63 c	8.31 ± 0.55 c	8262.07 ± 88.98 a	91.69 ± 0.55 a
2022–2023	FP	7418.22 ± 61.20 c	2431.58 ± 72.08 b	32.79 ± 1.24 a	4986.64 ± 133.28 b	67.21 ± 1.24 c
	HP	9489.69 ± 113.30 a	2581.58 ± 117.70 a	27.22 ± 1.55 b	6908.11 ± 230.99 a	72.78 ± 1.55 b
	OP	9086.09 ± 60.67 b	2110.97 ± 62.99 c	23.24 ± 0.85 c	6975.12 ± 123.65 a	76.76 ± 0.85 a
*p*-value						
Year (Y)		0.0062	0.0001	0.0001	0.0001	0.0001
Treatment (T)		0.0001	0.0001	0.0001	0.0001	0.0001
Y × T		0.7778	0.0148	0.0392	0.1765	0.0392

FP, local farmer’s agronomic practice; HP, high-input agronomic practice; OP, optimized high-input agronomic practice. Values followed by different letters within a column in the same year are significantly different at *p* < 0.05.

**Table 3:** N accumulation in grains and the contribution of pre-anthesis N remobilization and post-anthesis N uptake under different treatments in 2021–2022 and 2022–2023.

Year	Treatment	N accumulation in Grains (kg ha^−1^)	Pre-Anthesis N Remobilization		Post-Anthesis N Uptake	
			Amount (kg ha^−1^)	Contribution Rate (%)	Amount (kg ha^−1^)	Contribution Rate (%)
2021–2022	FP	159.45 ± 2.27 c	144.34 ± 3.19 c	90.53 ± 1.78 a	15.11 ± 2.88 c	9.47 ± 1.78 c
	HP	223.10 ± 3.05 a	191.21 ± 2.66 a	85.72 ± 1.97 b	31.89 ± 4.76 b	14.28 ± 1.97 b
	OP	206.55 ± 3.21 b	167.49 ± 2.37 b	81.11 ± 1.94 c	39.06 ± 4.51 a	18.89 ± 1.94 a
2022–2023	FP	173.22 ± 0.03 c	162.43 ± 3.68 c	93.77 ± 2.11 a	10.78 ± 3.65 b	6.23 ± 2.11 c
	HP	240.47 ± 1.76 a	210.93 ± 4.14 a	87.71 ± 1.16 b	29.54 ± 2.63 a	12.29 ± 1.16 b
	OP	216.95 ± 1.47 b	184.52 ± 3.58 b	85.05 ± 1.31 c	32.42 ± 2.76 a	14.95 ± 1.31 a
*p*-value						
Year (Y)		0.0001	0.0001	0.0004	0.0078	0.0004
Treatment (T)		0.0001	0.0001	0.0001	0.0001	0.0001
Y × T		0.0205	0.7229	0.5382	0.5112	0.5384

FP, local farmer’s agronomic practice; HP, high-input agronomic practice; OP, optimized high-input agronomic practice. Values followed by different letters within a column in the same year are significantly different at *p* < 0.05.

**Table 4:** The sowing date, planting density, and fertilization and irrigation management used under three integrated agronomic practice treatments.

Treatment	Sowing Date (m/d)	Planting Density (Seeds m^−2^)	Fertilization				Irrigation	
			Fertilizer	Pre-Sowing Amount (kg ha^−1^)	Topdressing Amount (kg ha^−1^)	Topdressing Timing	Frequency and Timing	Amount (mm)
FP	10/5	225	N	189	126	Regreening	5 times (Post-sowing, Before winter, Regreening, Anthesis, and Mid-filling)	350
			P_2_O_5_	120	−	−		
			K_2_O	30	−	−		
HP	10/10	375	N	126	189	Jointing	4 times (Post-sowing, Jointing, Anthesis, and Mid-filling)	280
			P_2_O_5_	210	−	−		
			K_2_O	150	−	−		
OP	10/15	450	N	96	144	Jointing	4 or 3 times (Post-sowing, Jointing, Anthesis, or Mid-filling)	280 or
			P_2_O_5_	120	−	−		210
			K_2_O	75	−	−		

FP, local farmer’s agronomic practice; HP, high-input agronomic practice; OP, optimized high-input agronomic practice. −, no data.

## Data Availability

The original contributions presented in the study are included in the article, further inquiries can be directed to the corresponding authors.

## Supplementary Materials

The following supporting information can be downloaded at: https://www.mdpi.com/article/10.3390/plants14203176/s1. Figure S1: Estimated marginal means for the significant Year × Treatment interaction on relevant indicators; Figure S2: Relative importance of variables affecting grain yield and water use efficiency (WUE).
