# Supplementary material for: Optimized High-Input Practice Enhances Wheat Productivity and Water Use Efficiency by Improving Root Distribution and Canopy Photosynthesis
Source: Plants (Basel). 2025 Oct 16;14(20):3176. doi: 10.3390/plants14203176 (PMC12567222; doi:10.3390/plants14203176)
Supplement: Supplementary file 1 [file plants-14-03176-s001.zip › plants-3905447-supplementary.pdf]

Supplementary Information

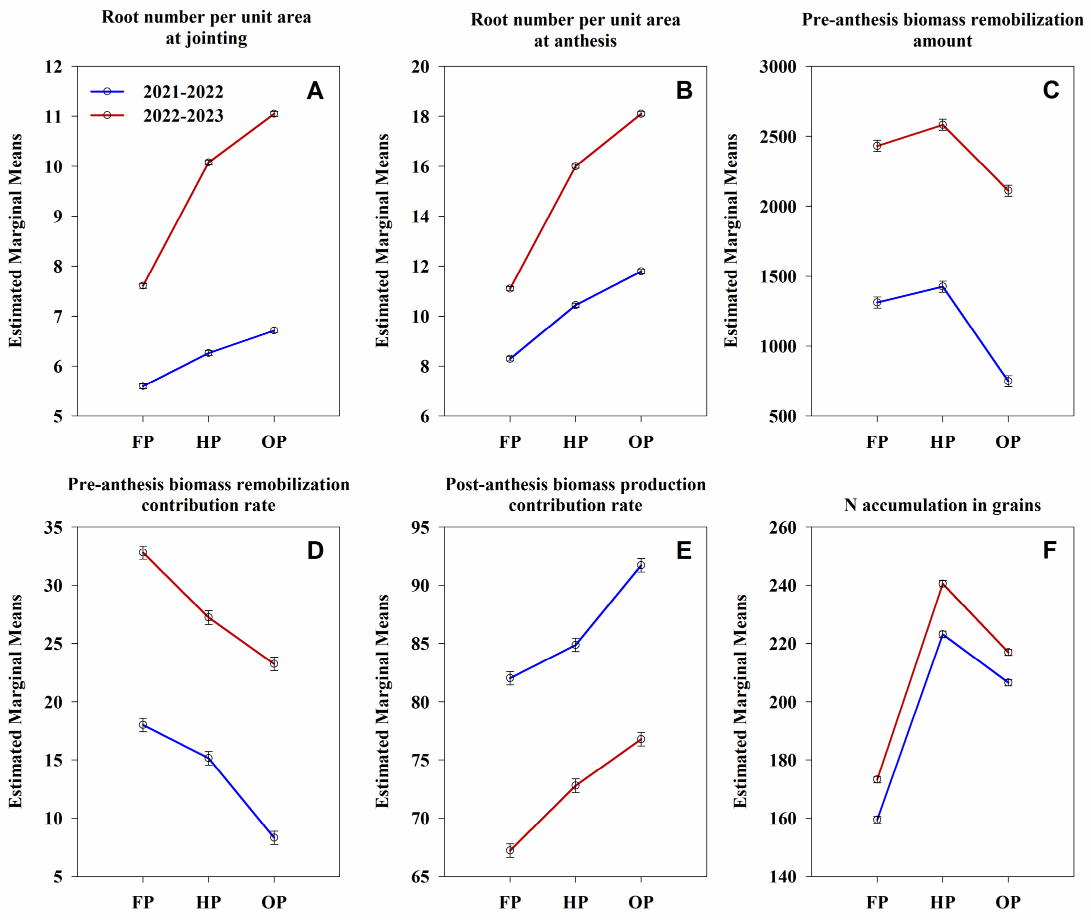

Figure S1. Estimated marginal means for the significant Year × Treatment interaction on relevant indicators. (**A**), root number per unit area at jointing; (**B**), root number per unit area at anthesis; (**C**), pre-anthesis biomass remobilization amount; (**D**), pre-anthesis biomass remobilization contribution rate; (**E**), Post-anthesis biomass production contribution rate; (**F**), N accumulation in grains.

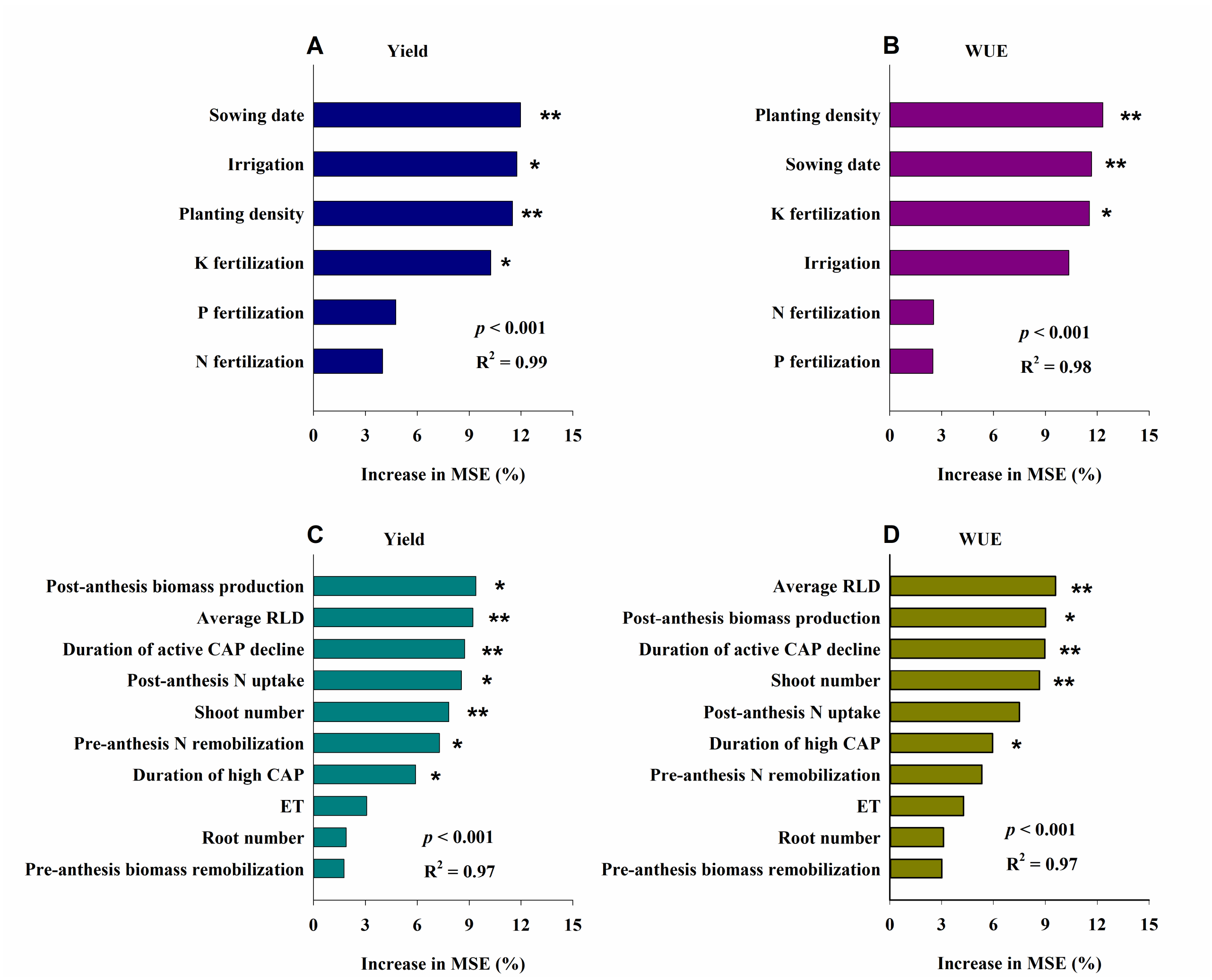

Figure S2. Relative importance of variables affecting grain yield and water use efficiency (WUE). (**A**,**B**), random forest mean predictor importance (% increase in mean square error (MSE)) of the agronomic practices as drivers of yield and WUE separately; (**C**,**D**), random forest mean predictor importance (% increase in MSE) of the studied physiological parameters as drivers of yield and WUE separately. Significance levels are shown at *$p < 0.05$ and **$p < 0.01$.
